# A CMOS Temperature Sensor with a Smart Calibrated Inaccuracy of ±0.11 (3σ)

**DOI:** 10.3390/s23115132

**Published:** 2023-05-27

**Authors:** Rongshan Wei, Huishan Lin, Qunchao Chen, Gongxing Huang, Wei Hu

**Affiliations:** College of Physics and Information Engineering, Fuzhou University, Fuzhou 350108, China; wrs08@fzu.edu.cn (R.W.); lhshaner@163.com (H.L.); qcchen@fzu.edu.cn (Q.C.); hgxwem01@163.com (G.H.)

**Keywords:** temperature sensor, delta-sigma modulation, calibration, neural network, high precision

## Abstract

This paper presents a BJT-based smart CMOS temperature sensor. The analog front-end circuit contains a bias circuit and a bipolar core; the data conversion interface features an incremental delta-sigma analog-to-digital converter. The circuit utilizes the chopping, correlated double sampling, and dynamic element matching techniques to mitigate the effects of process bias and nonideal device characteristics on measurement accuracy. Furthermore, based on the principle of charge conservation, the dynamic range utilization of the ADC increases. We propose a neural network that uses a multilayer convolutional perceptron to calibrate the sensor output results. Using the algorithm, the sensor achieves an inaccuracy of ±0.11 °C (3σ), exceeding the accuracy of ±0.23 °C (3σ) achieved without calibration. We implement the sensor in a 0.18 µm CMOS process, occupying an area of 0.42 mm^2^. It achieves a resolution of 0.01 °C and has a conversion time of 24 ms.

## 1. Introduction

Temperature sensors are widely applied in instrumentation acquisition, environmental monitoring, and overheating protection, among other applications. With the increasing number and diversity of applications, the requirements for sensors have become increasingly stringent. Therefore, temperature sensors that generate a readily interpretable digital output format without requiring additional configuration circuitry are significant. Such sensors are predominantly constructed using cost-effective standard CMOS processes that enable the integration of the analog front end (FEA), data conversion, and interface circuitry onto a single chip.

Several temperature-sensing mechanisms have been widely used in CMOS-integrated temperature sensors, such as thermistor-based [1], frequency quantization-based [2], and time-to-digital converter-based sensor mechanisms [3]. However, sensors based on bipolar junction transistors (BJTs) offer a robust solution and high accuracy after one-point calibration [4,5,6]. 

In recent years, a range of temperature sensors based on BJTs have emerged, employing various readout interfaces to enhance energy efficiency. These interfaces include Continuous Time (CT) delta-sigma ADC [7], SAR ADC [8], and zoom ADC [9]. However, when considering factors such as clock jitter, circuit complexity, and area, the Discrete Time (DT) delta-sigma ADC combines the aforementioned advantages.

Furthermore, the accuracy achievable by bipolar transistors is ultimately limited by variations in base-emitter voltage resulting from process scaling and mechanical stress. To address these limitations, trimming and calibration procedures are necessary to compensate for such variations. In addition to single-point calibration, alternative approaches like two-point calibration and heater-assisted voltage calibration have been proposed [10,11,12]. However, for applications requiring high accuracy, the conventional calibration techniques described above tend to be time consuming and, consequently, expensive.

This paper presents a BJT-based smart CMOS temperature sensor that operates over a temperature range of −45 °C to 125 °C. The sensor uses chopping, correlated double sampling (CDS), and dynamic element matching (DEM) [13] to minimize errors caused by op-amp, transistor, and current mirror mismatches. Additionally, proportional capacitive amplification is used to improve the usage of the incremental delta-sigma analog-to-digital converter (ADC) dynamic range. The digital curvature compensation mitigates nonlinearity in the transistor base and emitter stages. The proposed calibration method uses a multilayer convolutional perceptron neural network to improve accuracy from ±0.23 °C (3σ) to ±0.11 °C (3σ) compared to that without calibration. The remainder of the paper is structured as follows: in Section 2, the topology of the sensor is described and the operating principle of BJT-based sensors is explained in brief. In Section 3, Section 4 and Section 5, the circuit and algorithm implementation is presented, followed by experimental results in Section 6. Finally, a summary of the conclusions drawn from this study is presented in Section 7.

## 2. Operating Principle

BJTs are suitable for ratio-metric measurement schemes [14], resulting in the generation of an accurate voltage proportional to absolute temperature (PTAT) and a temperature-independent bandgap reference voltage. Herein, a digital circuit is used to assist the analog circuit for implementing the temperature sensor. Using digital circuitry for some complex circuit functions provides area and power consumption benefits while simplifying nonlinear compensation and calibration.

Pertijs et al. [5] proposed a BJT-based temperature sensor that uses a custom delta-sigma ADC. Instead of generating a temperature-independent reference voltage as the ADC reference, the sensor dynamically adjusts *V_BE_* and ∆*V_BE_* based on the ADC output, indirectly generating the desired quantized output result *μ*. Equations (1) and (2) express this process.

(1) $$(1-\mu )\cdot \alpha \cdot \Delta V_{BE}=\mu \cdot V_{BE}$$

(2) $$\mu =\frac{\alpha \cdot \Delta V_{\mathrm{BE}}}{V_{\mathrm{BE}}+\alpha \cdot \Delta V_{\mathrm{BE}}}=\frac{\alpha \cdot \Delta V_{\mathrm{BE}}}{V_{\mathrm{REF}}}$$

The proposed scheme aims to address the issue of temperature curvature compensation by adjusting the ratio of *V_BE_* and ∆*V_BE_*. Although this approach can offer benefits, the practical implementation of the scaling factor α remains challenging.

**Figure 1:**
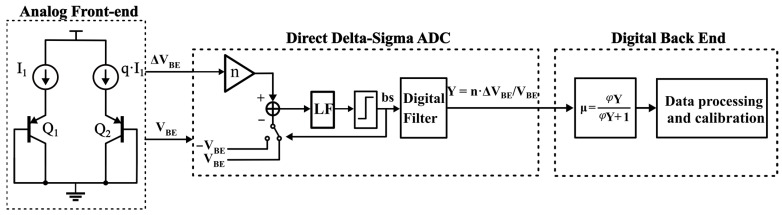
Block diagram of the temperature sensor.

This study uses a temperature sensor, which utilizes ∆*V_BE_* voltage as the input signal and *V_BE_* voltage as the quantized reference voltage for the delta-sigma ADC, as shown in Figure 1. The output of the delta-sigma ADC is ∆*V_BE_/V_BE_*, which can be further processed through digital circuitry to obtain the desired temperature signal *μ* of the PTAT. The expression for the temperature signal *μ* is given as follows:

(3) $$\mu =\frac{\frac{\Delta V_{BE}}{V_{BE}}}{1+\alpha \cdot \frac{\Delta V_{BE}}{V_{BE}}}$$

The above-described design offers flexibility in designing the scale factor α in digital circuits. However, the maximum quantization result of this method is approximately 0.1 at temperatures ranging between −45 °C and 125 °C. This limitation results in insufficient utilization of the ADC dynamic range.

The design incorporates a fourfold amplified ∆*V_BE_* voltage as the input to the delta-sigma ADC to enhance the utilization of the ADC dynamic range. Therefore, we recorded the quantization result of the ADC as *Y* = 4·∆*V_BE_*/*V_BE_*. Subsequently, we can express the equation for the temperature-dependent signal *μ* as follows:

(4) $$\mu =\frac{\alpha \cdot \Delta V_{\mathrm{BE}}}{V_{\mathrm{BE}}+\alpha \cdot \Delta V_{\mathrm{BE}}}=\frac{\varphi Y}{\varphi Y+1}$$

where the constant φ has a value of *α*/4, and Equation (4) can be readily implemented in digital circuits. 

**Figure 2:**
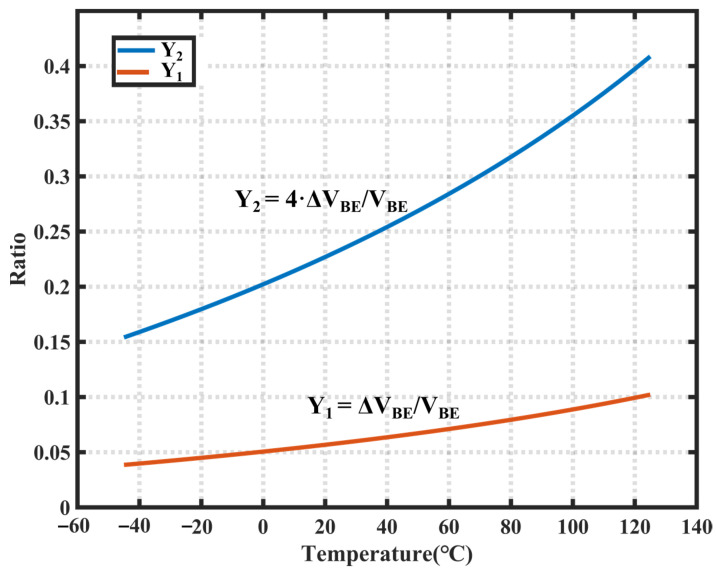
Dynamic range utilization of ADC.

As shown in Figure 2, the use of *Y* = 4·∆*V_BE_/V_BE_* allows for greater utilization of the dynamic range of the ADC.

## 3. Analog Front-End Circuits

**Figure 3:**
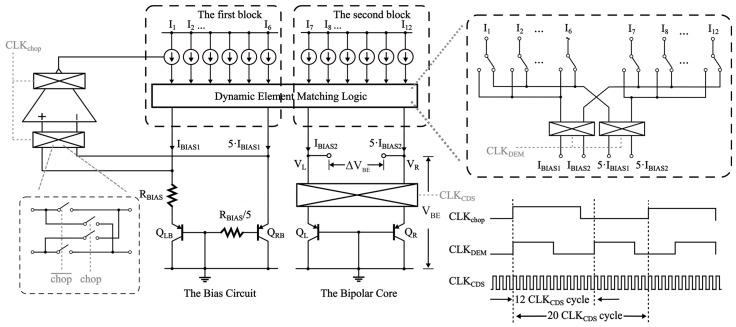
BJT-Based CMOS temperature sensor front-end circuit.

The analog front-end circuit used herein, as illustrated in Figure 3, comprises two major parts: the bias circuit and the bipolar core [15]. Two substrate PNPs, Q_LB_ and Q_RB_, biased by different currents, generate two voltage signals, *V_BEbias2_* and *V_BEbias1_*, respectively, and a ∆*V_BEbias_* voltage in the primary circuit [16]. These signals can be expressed as follows:

(5) $$\Delta V_{\mathrm{BEbias}}=V_{\mathrm{BEbias}2}-V_{\mathrm{BEbias}1}=\varepsilon \cdot \frac{kT}{q}\ln\left(\frac{pI_{C}}{I_{S}}\right)-\varepsilon \cdot \frac{kT}{q}\ln\left(\frac{I_{C}}{I_{S}}\right)=\varepsilon \cdot \frac{kT}{q}\ln\left(p\right)$$

where the coefficient *p* is the ratio of the collector current densities of the two BJT tubes, and *I_C_* represents the collector current. Additionally, it can be derived that Δ*V_BEbias_* is PTAT, where ε is the process-dependent nonlinearity factor, *k* is Boltzmann’s constant, *q* represents the electron charge, and *T* is the absolute temperature.

The op-amp establishes a feedback loop in the bias circuit, clamping the input terminal voltages to be equal, allowing it to operate. This results in the application of Δ*V_BEbias_* across the bias resistor *R_BIAS_*, thus generating the *I_BIAS_* necessary for the circuit’s operation.

(6) $$I_{\mathrm{BIAS}\;}=\frac{\Delta V_{\mathrm{BEbias}}}{R_{\mathrm{BIAS}\;}}=\frac{\varepsilon \cdot kT}{q\cdot R_{\mathrm{BIAS}\;}}\ln\left(p\right)$$

The bias circuit produces *I_BIAS_*, which is then replicated through the current mirror and delivered to the triode in the bipolar core. This leads to the generation of the ∆*V_BE_* voltage and the *V_BE_* voltage, which together form the temperature signal and serve as the ADC input signal and the reference voltage, respectively. 

### 3.1. Bias Current Design

The bipolar transistor is connected as a diode to reduce base current leakage. This connection results in a change in the relationship between the current gain and transistor currents, which can be expressed as follows:

(7) $$p_{C}=\frac{I_{C2}}{I_{C1}}=\frac{1+\beta_{1}}{\beta_{1}}\cdot \frac{\beta_{2}}{1+\beta_{2}}\cdot \frac{I_{E2}}{I_{E1}}=\frac{1+\beta_{1}}{\beta_{1}}\cdot \frac{\beta_{2}}{1+\beta_{2}}\cdot p$$

where *I_C1_* and *I_C2_* denote the collector currents of different bipolar transistors, *I_E1_* and *I_E2_* represent the emitter currents, and *β_1_* and *β_2_* are the current gains. Additionally, the collector and emitter current ratios of the two substrate PNPs are denoted as *p_c_* and *p*.

**Figure 4:**
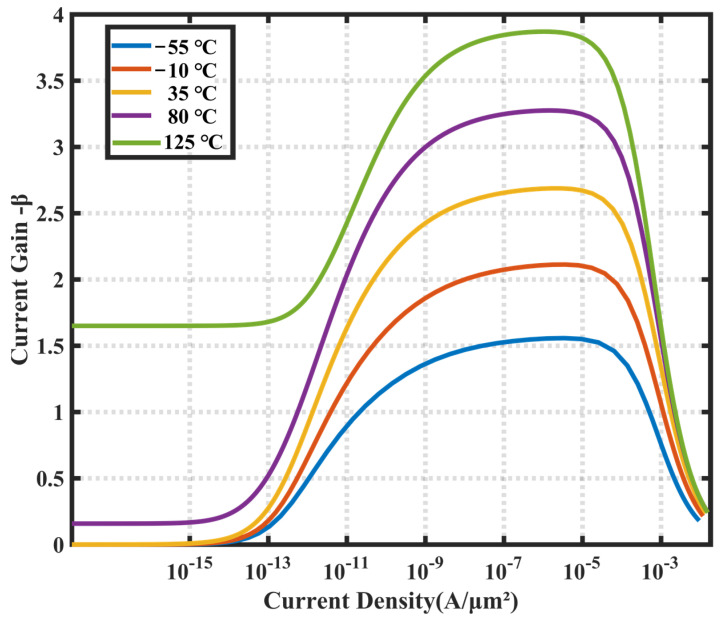
Variation of *β* with *I_C_* current density at different temperatures.

Ensuring that *β_1_* and *β_2_* are equal is necessary to maintain a stable *I_E_* bias ratio. This subsequently requires a high level of stability in *β* over the set current operating range. As shown in Figure 4, *β* varies with *I_C_* current density at different temperatures in the 0.18 µm CMOS process.

There is only a slight variation in the value of *β* within the temperature range of −55 °C to 125 °C in this process, with the value ranging from 1.5 to 4.

Selecting appropriate values for the current density ratio (*p*) and bias current (*I_BIAS_*) involves a comprehensive evaluation of multiple factors, including noise, capacitive load driving capability, and circuit robustness. The bias currents of the two substrate PNPs in the biased circuit are 1.8 and 9 μA, respectively, resulting in an area ratio of 2:1 and a current density ratio (*p*) of 10. The bias current of the two substrate PNPs in the bipolar core was set to the same level as that of the bias circuit. Consequently, the transistors have equal areas with a ratio of 1:1 and a current density ratio (*p*) of five. Additionally, the requirements of the layout design must be considered while selecting the number of BJTs to minimize mismatch errors. The finite value of *β* in this process will cause significant inaccuracies in the output results. Therefore, a resistor with a value of *R_BIAS_*/m is connected in series with the base of the high-current biased BJT to make the *V_BEbias_* independent of the *β* value, eliminating the effect of limited current gain.

### 3.2. DEM

The primary source of error in ∆*V_BE_* is a current ratio mismatch between the two current branches [16]. For example, assuming a current density ratio mismatch of ∆*p*, the absolute error in ∆*V_BE_* can be expressed as follows:

(8) $$\Delta V_{BE}-\Delta V_{BE}{}_{|\Delta p=0}\approx \varepsilon \cdot \frac{kT}{q}\ln\left(p\right)\cdot \left(1+\frac{\Delta p}{p\ln p}\right)-\varepsilon \cdot \frac{kT}{q}\ln\left(p\right)\approx \varepsilon \cdot \frac{kT}{q}\cdot \frac{\Delta p}{p}$$

The error in the ∆*V_BE_* resulting from current density mismatch is observed to be proportional to absolute temperature. Therefore, a precision of ∆*p*/*p* ≈ 0.1% can be achieved by carefully designing the layout of the current sources [17]. To further enhance the accuracy of the current source in the analog front-end circuit, the ALL-DEM [18] approach is employed herein for dynamic element matching of the current mirror. The fundamental principle of this approach is to dynamically swap circuit devices to average device mismatch, reducing the overall mismatch between devices.

The bias circuit and bipolar core contain two blocks, each with six-unit current sources, to ensure DEM matching. In the φ1 phase of the sampling period, the left bipolar transistor in each block is biased by a unit current source selected in turn while the remaining five-unit current sources are used to bias the suitable bipolar transistor. In the second phase (φ2) of the sampling period, the bipolar core uses double CDS to exchange the PNP bias, creating a fresh set of *V_BE_* and ∆*V_BE_* voltages. This process is intended to mitigate the mismatch of bipolar transistors and assumes the following steps to eliminate area mismatch (∆*r*) of a bipolar transistor:

(9) $$\begin{array}{l} \Delta V_{BEA}-\Delta V_{BEB}\\ =\varepsilon \cdot \frac{kT}{q}\ln\left(\frac{p+\Delta p}{1+\Delta r}\right)-(\varepsilon \cdot \frac{kT}{q}\ln\left(\left(p+\Delta p\right)\left(1+\Delta r\right)\right)\\ =2\cdot \varepsilon \cdot \frac{kT}{q}\ln\left(p+\Delta p\right), \end{array}$$

where ∆*V_BEA_* and ∆*V_BEB_* are generated before and after CDS, respectively; taking the difference between these two ∆*V_BE_s* entirely cancels the error caused by ∆*r*. This process is repeated during the next sampling period until both the current mirrors of the first and the second blocks complete one cycle of biasing the biased circuit and the bipolar core, following which the contemporary mirrors of the first and second blocks are swapped, and the cycle continues in a loop. Averaging the ∆*V_BE_* voltage eliminates most of the error, leaving only high-order errors. The error obtained following DEM can be mathematically expressed as

(10) $$\left|\Delta V_{BE,avg}-\Delta V_{BE|\Delta p=0}\right|<\varepsilon \cdot \frac{1}{2}\cdot \frac{kT}{q}\cdot {\left(\frac{\Delta p}{p}\right)}^{2}$$

## 4. Incremental Delta-Sigma Modulator 

### 4.1. Modulator Topology

**Figure 5:**
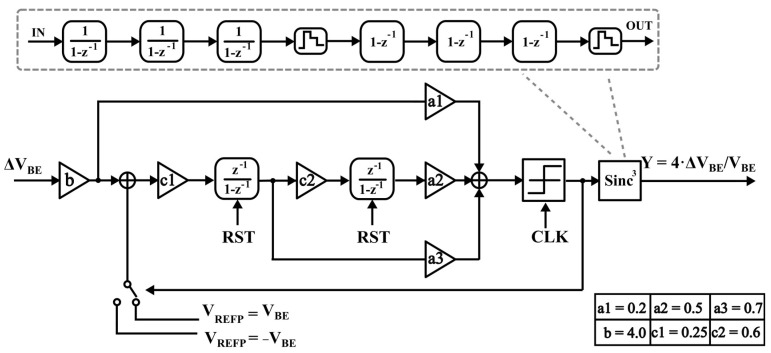
Incremental delta-sigma ADC topologies.

The ADC presented herein comprises an incremental delta-sigma modulator and a Sinc^3^ digital filter [19]. The modulator is implemented using a second-order single-bit CIFF structure; its topology is shown in Figure 5. 

The effective number of bits (ENOB) for the incremental delta-sigma ADC using this structure can be mathematically expressed as [20]:

(11) $$ENOB_{2st,ideal}\approx 2\cdot \log_{2}\left(N\right)+\log_{2}\left(bc_{1}c_{2}\right)-1$$

Considering the limited OTA gain, digital filter type, and other relevant factors, this paper has chosen a conversion period of N = 600 and a sampling clock frequency of f = 25 kHz.

### 4.2. Modulator Circuit Implementation

**Figure 6:**
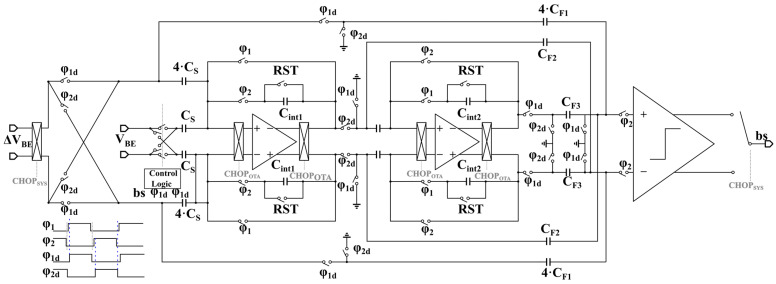
Simplified circuit diagram of the proposed incremental delta-sigma modulator.

**Figure 7:**
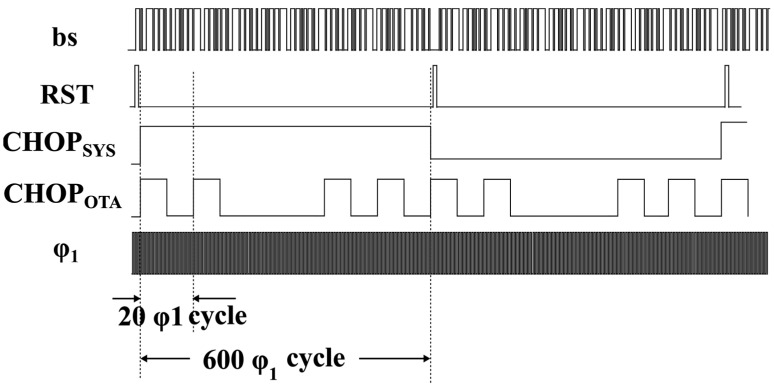
Timing diagram of the incremental delta-sigma modulator.

The circuit block diagram and timing diagram of the incremental delta-sigma modulator are presented in Figure 6 and Figure 7. To enhance the dynamic range utilization of the incremental delta-sigma modulator, the capacitance ratio of the ∆*V_BE_* sampling capacitor is set to four times that of the *V_BE_* sampling capacitor, achieving ∆*V_BE_* amplification [21]. Consequently, the charge acquired from the sampling process during one sampling period can be mathematically expressed as

(12) $$Q_{\Delta V_{BE}}=4\cdot C_{S}\cdot \left(2\cdot \Delta V_{BE}\right)$$

Multiplication by two accounts for double sampling. While the feedback voltage is sampled using a unit sampling capacitor, the resulting charge can be expressed as

(13) $$Q_{V_{REF}}=C_{S}\cdot \left(2\cdot V_{REF}\right)$$

The incremental delta-sigma modulator maintains charge balance through negative feedback control by setting the reference voltage to *V_BE_* and maintaining the average charge at zero. The following expression can thus be derived:

(14) $$Y\cdot 2C_{S}\cdot \left(4\Delta V_{BE}-V_{BE}\right)+\left(1-Y\right)\cdot 2C_{S}\cdot \left(4\Delta V_{BE}+V_{BE}\right)=0$$

The output *Y* of the ADC can be obtained using the following equation, where *Y* represents the average value of the code stream output from ADC:

(15) $$Y=\frac{4\cdot \Delta V_{BE}}{V_{BE}}$$

To mitigate the impact of nonideal factors such as detuning and noise on the quantization accuracy of the delta-sigma ADC, circuit design techniques such as autozeroing and chopping techniques are used herein because the temperature signal is a low-frequency signal closely related to DC and is significantly affected by DC detuning and flicker noise.

## 5. Multilayer Convolutional Perceptron Calibration Network

To further reduce sensor errors, this study proposes a multilayer convolutional perceptron (MLCP) neural network algorithm that leverages one-dimensional convolution (Conv1d) within the hidden layer to extract relevant features based on the principles of a multilayer perceptron [22]. The MLCP architecture proposed herein is as follows.

**Figure 8:**
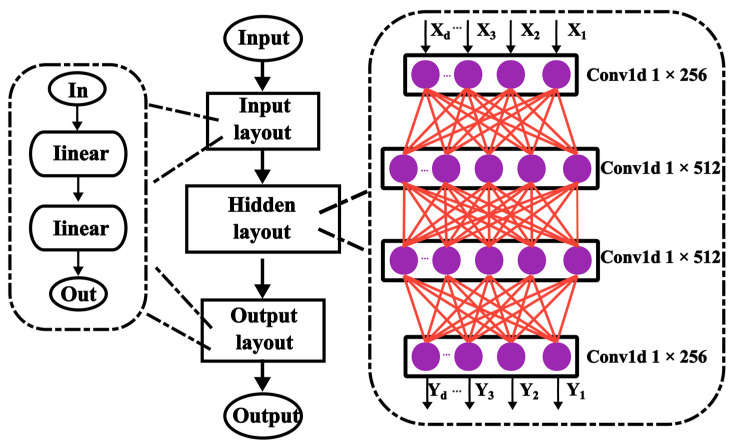
MLCP neural network structure.

As shown in Figure 8, the MLCP network, similar to BP neural networks [23], comprises three distinct components: the input, hidden, and output layers. The input and output layers include two linear layers while the hidden layer comprises four Conv1d layers. First, the sensor data are fed into the network via the input layer and processed by the Conv1d hidden layer to extract relevant features. Then, linear variation preprocessing is applied at the input layer to improve the subsequent network processing and fitting capability of the model. The linear variation equation is given as follows:

(16) $$X=V\times A^{T}+B$$

where *X* represents the output, *V* represents the input, and *A* and *B* represent the weight and bias matrices, respectively. Two linear variations in preprocessing were applied to the information to consider the calibration temperature and alignment with subsequent network nodes. The formula for Conv1d is given as follows:

(17) $$\mathrm{out}\left(N_{i},C_{{\mathrm{out}\;}_{j}}\right)=\mathrm{bias}\left(C_{{\mathrm{out}\;}_{j}}\right)+\sum_{k=0}^{C_{\mathrm{in}\;}-1}weight\left(C_{{\mathrm{out}\;}_{j}},k\right)*\mathrm{input}\left(N_{i},k\right)$$

where * denotes the valid cross-correlation operator, *N* denotes batch size, *C* denotes the number of channels, *L* denotes the length of the signal sequence, and *k* denotes the convolution depth. For the dataset considered here, *L* = 1 and *N* = 1. The convolution kernels have a size of one with no padding and a stride of one. Because of the characteristics of the temperature sensor output data, using one-dimensional convolution in the hidden layer can improve accuracy and enable better learning of the correlation information between each node. Finally, the output layer uses the processed input sensor data to generate the final output.

We used Monte Carlo simulations to generate the output results of 1000 sensors within a temperature range of −45 °C to 125 °C. We used the output data of 100 sensors with the same temperature as that in the input during training. The epoch was set to 1000 rounds. The learning rate was reduced from $10^{-3}$ to $10^{-10}$ using the cosine annealing strategy. The number of learning rate rounds for warming up was three, during which the learning rate was maintained constant. The Adam optimizer was used to optimize loss values, where the loss function was mean squared error (MSE) loss, which was calculated as follows:

(18) $$\ell (x,y)=\frac{{\left(x_{1}-y_{1}\right)}^{2}+{\left(x_{2}-y_{2}\right)}^{2}+\ldots +{\left(x_{n}-y_{n}\right)}^{2}}{n}$$

where *x* represents the input and *y* denotes the target.

## 6. Experimental Results

The temperature sensor was implemented using a 0.18 µm CMOS process with six metal layers, resulting in an active area of 0.422 ${\mathrm{mm}}^{2}$. Furthermore, to increase flexibility, the decimation filter and digital back end were implemented off chip. The sensor operates at 1.8 V supply voltage and a 25 kHz clock frequency, consuming a current of 201 µA. It takes 24 ms to complete 600 measurement cycles. 

**Figure 9:**
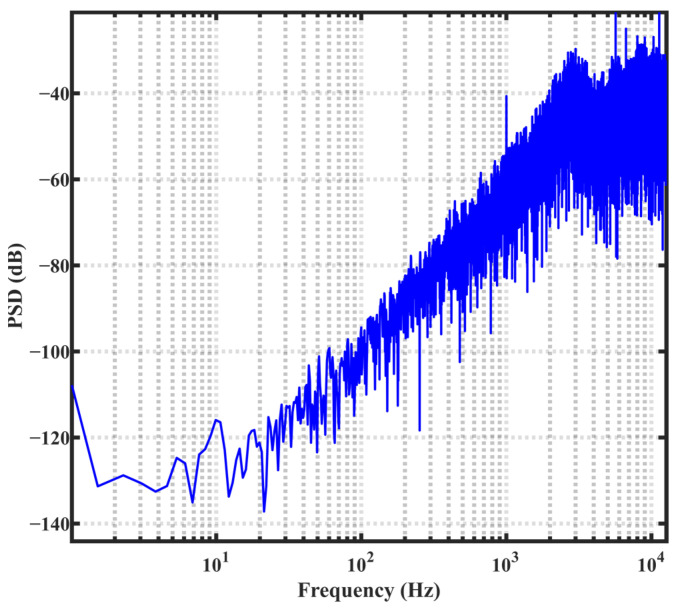
Power spectrum of the modulator.

**Figure 10:**
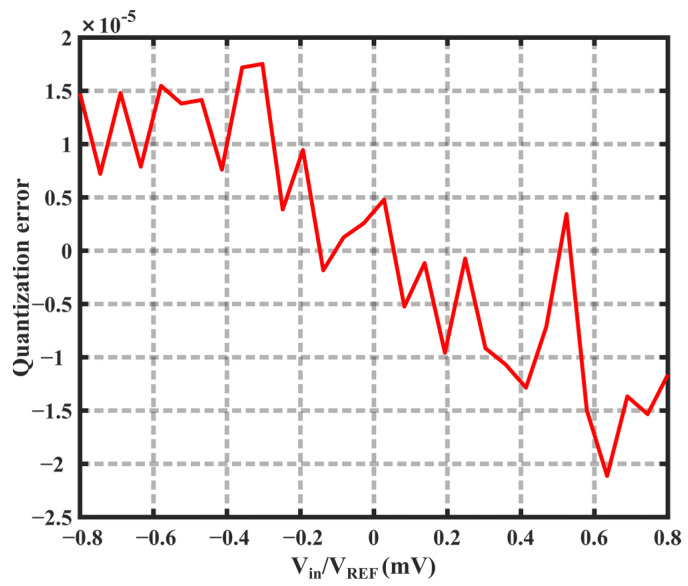
Quantization error of the modulator.

**Figure 11:**
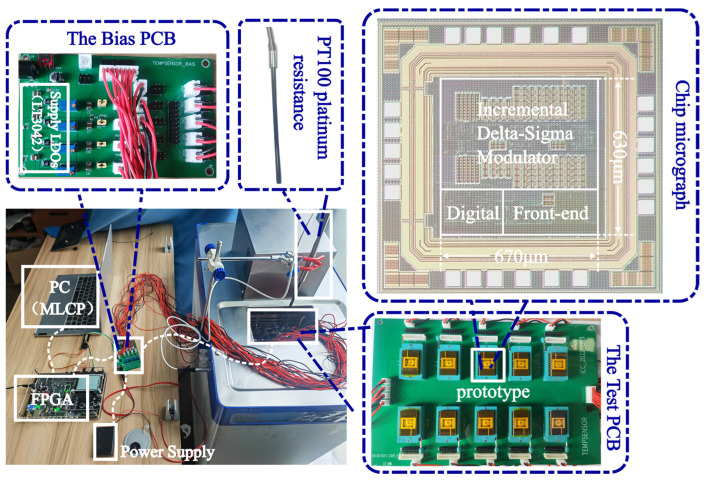
Setup of the temperature sensor test.

Figure 9 illustrates the power spectrum of the bitstream of the modulator, highlighting its effective noise-shaping capability [24]. Figure 10 shows the quantization error of the same ADC. During the sensor test, a high-precision thermostat tank filled with silicone oil was utilized to maintain a constant temperature in the test environment. Furthermore, a high-precision PT100 platinum resistor served as the reference temperature sensor. Figure 11 depicts the sensor test setup and chip micrographs.

**Figure 12:**
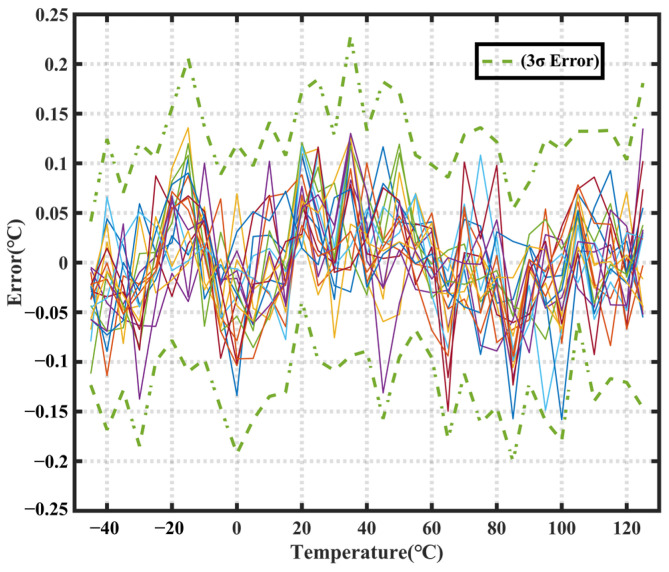
Temperature sensor test results obtained without calibration.

To determine the temperature error of the sensors, 18 sensors from a single batch were mounted in a dual in-line package (DIP) and measured over a temperature range of −45 °C to 125 °C. Figure 12 shows the temperature error of the 18 samples before trimming. The 3σ spread over the range of 45 °C to 125 °C is 0.23 °C.

**Figure 13:**
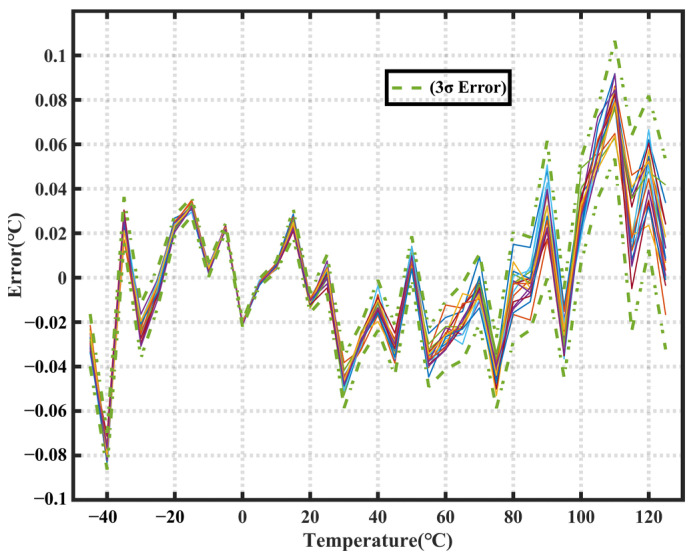
Temperature sensor test results with a calibration MLCP neural network algorithm.

To enhance the accuracy of the sensor, we used the MLCP neural network algorithm for sensor calibration, which yielded the calibration results plotted in Figure 13.

**Figure 14:**
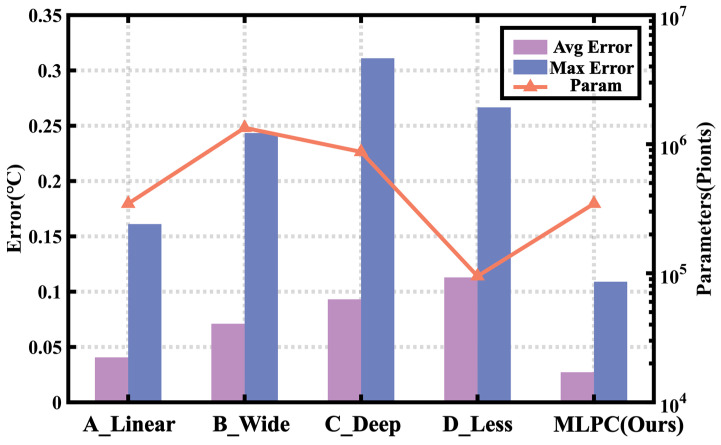
Effectiveness and cost comparison of different algorithm models.

As shown in Figure 13, the maximum error decreased from 0.23 °C (3σ) to 0.11 °C (3σ), with a maximum error of less than 0.06 °C within the commonly used temperature range of 0 to 100 °C, thereby validating the effectiveness of the MLCP neural network model. To further evaluate the performance of the MLCP model, four additional models were implemented in this study: A_Linear, which replaced the hidden layer with a linear connection; B_Wide, which doubled the dimensionality in the hidden layer; C_Deep, which added two extra hidden layers; and D_Less, which doubled the nodes in the output layer. All models were trained and tested in the same environment, and the final results are presented in Figure 14.

Figure 14 shows that, despite having fewer parameters (Param) than other algorithms, the developed model achieves the highest accuracy and has a significantly low maximum error. This result showcases the effectiveness of the proposed model design.

Table 1 summarizes the performance of the proposed model and compares it to other temperature sensor technologies. Reference [25] presents a favorable cost advantage attributed to its small area; however, it necessitates the utilization of a high-frequency clock (fs = 20 MHz) for proper operation, which imposes limitations on its application environment. References [26,27] employ BJTs as the means to generate PTAT voltages, subsequently utilizing ADC acquisition for temperature conversion. However, this approach exhibits certain drawbacks in terms of resolution and accuracy when compared to the methodology proposed in this paper. Reference [12] presents notable advantages in terms of power consumption and accuracy. However, the inherent complexity of its circuit structure and the necessity for a third-order fit in obtaining results pose significant challenges in terms of implementation complexity and cost. The temperature sensor achieves a resolution of over 0.01 °C and consumes 275.4 µW of power at a 1.8 V supply. Its resolution FOM is 661 p. The sensor boasts impressive resolution and accuracy, albeit at a higher power consumption level than other sensors. Furthermore, its accuracy is as good as ±0.06 °C (3σ) within the commonly used temperature measurement range.

## 7. Conclusions

The BJT-based smart calibration temperature sensor was designed and verified in a 0.18 µm CMOS process. The sensor mitigates the mismatch of the current mirror cell and the BJT through ALL-DEM and CDS techniques, while reducing the sensor’s inaccuracy caused by offset voltage of amplifier and ADC through the utilization of the chopping technique. Additionally, charge conservation is utilized to improve the utilization of the ADC dynamic range, thereby enhancing the system’s robustness. To calibrate the output data and enhance the sensor’s accuracy, the MLCP neural network algorithm is proposed in this paper, which reduces the sensor’s inaccuracy from ±0.23 °C (3σ) to 0.11 °C (3σ) across a temperature range of −45 °C to 125 °C. Within the commonly used temperature measurement range of −35 °C to 100 °C, the accuracy is as good as ±0.06 °C (3σ). The sensor has an effective area of 0.42 mm^2^, and the conversion time of the sensor is 24 ms.

## Figures and Tables

**Table 1:** Performance summary and comparison of temperature sensors.

Parameters	[25]	[26]	[12]	[27]	This Work	
Tech (µm)	0.13	0.13	0.16	0.18	0.18	
Area (mm^2^)	0.086	0.29	0.15	0.13	0.42	
VDD (V)	1.2/3.3	2-3.6	1.8	1.8/3.3	1.8	
Power (µW)	39.7	313.5	9.75	45.7	275.4	
Temperature Range (°C)	−40 to 125	−40 to 125	−40 to 180	−50 to 130	−45 to 125	−35 to 100
Resolution (°C)	0.06	0.016	0.023	0.04	0.01	
Inaccuracy (°C)	±0.54 (3σ)	±0.47 (3σ)	±0.2 (3σ)	±0.8 (3σ)	±0.11 (3σ)	±0.06 (3σ)
Conv. Time (ms)	1.3	5.12	20	10.24	24	
Rel. InAcc (%) **	0.655	0.57	0.18	0.8	0.129	
Res. FoM (pJ°C^2^) *	186	411	103	749	661	

* Res. FoM = Energy/Conversion × Resolution^2^; ** Rel. InAcc = 2·Inaccuracy/Range × 100.

## Data Availability

Not applicable.

## Abbreviations

The following abbreviations are used in this manuscript:

ADC	Analog-to-digital converter
CT	Continuous Time
DT	Discrete Time
CDS	Corelated double sampling
BJT	Bipolar junction transistors
DEM	Dynamic element matching
PTAT	Proportional to absolute temperature
OTA	Operational transconductance amplifier
MLCP	Multilayer convolutional perceptron
Conv1d	One-dimensional convolution
ENOB	effective number of bits

## References

[1] Pan S., Makinwa K.A. (2021). A 10 fJ·K2 Wheatstone Bridge Temperature Sensor With a Tail-Resistor-Linearized OTA. IEEE J. Solid-State Circuits.

[2] Perrott M., Salvia J., Lee F., Partridge A., Mukherjee S., Arft C., Kim J.T., Arumugam N., Gupta P., Tabatabaei S. A temperature-to-digital converter for a MEMS-based programmable oscillator with better than ±0.5 ppm frequency stability. Proceedings of the 2012 IEEE International Solid-State Circuits Conference.

[3] Lin S.J., Chen H.C. (2022). A TDC-Based Temperature Sensor for Biomedical Applications. IEEE Sens. J..

[4] Souri K., Makinwa K. A 0.12 mm^2^ 7.4 μW micropower temperature sensor with an inaccuracy of ±0.2 °C (3σ) from −30 °C to 125 °C. Proceedings of the 36th European Solid State Circuits Conference (ESSCIRC 2010).

[5] Pertijs M.A.P., Niederkorn A., Xu M., McKillop B., Bakker A., Huijsing J.H. (2005). A CMOS smart temperature sensor with a 3/spl sigma/ inaccuracy of /spl plusmn/0.5/spl deg/C from −50/spl deg/C to 120/spl deg/C. IEEE J. Solid-State Circuits.

[6] Pertijs M.A.P., Makinwa K.A.A., Huijsing J.H. (2005). A CMOS smart temperature sensor with a 3/spl sigma/ inaccuracy of /spl plusmn/0.1/spl deg/C from −55/spl deg/C to 125/spl deg/C. IEEE J. Solid-State Circuits.

[7] Yousefzadeh B., Shalmany S.H., Makinwa K.A.A. (2017). A BJT-Based Temperature-to-Digital Converter With ±60 mK (3σ) Inaccuracy From −55 °C to +125 °C in 0.16-μm CMOS. IEEE J. Solid-State Circuits.

[8] Ku H.S., Choi S., Sim J.Y. (2022). A 12μs-Conversion, 20mK-Resolution Temperature Sensor Based on SAR ADC. IEEE Trans. Circuits Syst. II: Express Briefs.

[9] Xie S., Ge X., Theuwissen A. Temperature sensors incorporated into a cmos image sensor with column zoom adcs. Proceedings of the 2019 IEEE International Symposium on Circuits and Systems (ISCAS).

[10] Banarie G., McDonagh D., Bucur V., Marinca S., Bodea M. A BJT BiCMOS Temperature Sensor with a Two-Point Calibrated Inaccuracy of 0.1 °C (3σ) from −40 to 125 °C. Proceedings of the 2018 29th Irish Signals and Systems Conference (ISSC).

[11] Yousefzadeh B., Makinwa K.A.A. 9.3 A BJT-based temperature sensor with a packaging-robust inaccuracy of ±0.3 °C (3σ) from −55 °C to +125 °C after heater-assisted voltage calibration. Proceedings of the 2017 IEEE International Solid-State Circuits Conference (ISSCC).

[12] Yousefzadeh B., Makinwa K.A.A. (2020). A BJT-Based Temperature-to-Digital Converter With a ±0.25 °C 3σ-Inaccuracy From −40 °C to +180 °C Using Heater-Assisted Voltage Calibration. IEEE J. Solid-State Circuits.

[13] van de Plassche R. Dynamic element matching for high-accuracy monolithic D/A converters. Proceedings of the 1976 IEEE International Solid-State Circuits Conference. Digest of Technical Papers.

[14] Meijer G.C.M. (1986). Thermal sensors based on transistors. Sens. Actuators.

[15] Klaassen K.B. (1975). Digitally Controlled Absolute Voltage Division. IEEE Trans. Instrum. Meas..

[16] Pertijs M.A.P., Huijsing J.H., Pertijs M.A.P., Huijsing J.H. (2006). Ratiometric temperature measurement using bipolar transistors. Precision Temperature Sensors in Cmos Technology.

[17] Hastings A. (2001). The Art of Analog Layout.

[18] Wei R., Bao X. (2018). A Low Power Energy-Efficient Precision CMOS Temperature Sensor †. Micromachines.

[19] Markus J., Silva J., Temes G.C. (2004). Theory and applications of incremental /spl Delta//spl Sigma/ converters. IEEE Trans. Circuits Syst. I Regul. Pap..

[20] Markus J., Silva J., Temes G.C. Design theory for high-order incremental converters. Proceedings of the IEEE International Symposium on Intelligent Signal Processing.

[21] Choi T., Kaneshiro R., Brodersen R., Gray P., Jett W., Wilcox M. High frequency CMOS switched capacitor filters for communication applications. Proceedings of the 1983 IEEE International Solid-State Circuits Conference. Digest of Technical Papers.

[22] Wei R., Ouyang K., Bao X., Gao X., Chen C. (2019). High-precision smart calibration system for temperature sensors. Sens. Actuators A Phys..

[23] Goh A.T.C. (1995). Back-propagation neural networks for modeling complex systems. Artif. Intell. Eng..

[24] Candy J. (1985). A Use of Double Integration in Sigma Delta Modulation. IEEE Trans. Commun..

[25] Huang Z., Tang Z., Yu X.-P., Shi Z., Lin L., Tan N. (2021). A BJT-based CMOS Temperature Sensor with Duty-cycle-modulated Output and ±0.54 °C (3σ) Inaccuracy from −40 °C to 125 °C. IEEE Trans. Circuits Syst. II Express Briefs.

[26] Tang Z., Fang Y., Yu X.P., Shi Z., Tan N. (2018). A CMOS Temperature Sensor With Versatile Readout Scheme and High Accuracy for Multi-Sensor Systems. IEEE Trans. Circuits Syst. I: Regul. Pap..

[27] Qin C., Huang Z., Liu Y., Li J., Lin L., Tan N., Yu X. (2022). An Energy-Efficient BJT-Based Temperature Sensor with ±0.8 °C (3σ) Inaccuracy from −50 to 150 °C. Sensors.

